# Understanding e-Cigarette Addictiveness: Triangulation of Focus Group and Netnographic Data

**DOI:** 10.2196/57970

**Published:** 2024-10-01

**Authors:** Marike Andreas, Nadja Grundinger, Nadine Wolber, Daria Szafran, Tatiana Görig, Ute Mons, Valerie Lohner, Sabine Vollstädt-Klein, Sven Schneider

**Affiliations:** 1 Division of Public Health, Social and Preventive Medicine Center for Preventive Medicine and Digital Health Medical Faculty Mannheim, Heidelberg University Mannheim Germany; 2 Department of Addictive Behavior and Addiction Medicine Central Institute of Mental Health Medical Faculty Mannheim, Heidelberg University Mannheim Germany; 3 Department of Medical Informatics, Biometry and Epidemiology Friedrich-Alexander-Universitat Erlangen-Nurnberg Erlangen Germany; 4 Cardiovascular Epidemiology of Aging, Department of Cardiology Faculty of Medicine and University Hospital Cologne University of Cologne Cologne Germany; 5 Mannheim Center for Translational Neurosciences Medical Faculty of Mannheim Heidelberg University Mannheim Germany

**Keywords:** e-cigarettes, online forums, netnographic analysis, addictive, addiction, smoking cessation, smoker, user, focus group, nicotine, public health, prevalence, smoking behavior

## Abstract

**Background:**

Numerous studies have shown that e-cigarettes are addictive. For example, we previously showed that users of e-cigarette online forums discuss experiences of addiction in a netnographic analysis. However, it is unclear what makes e-cigarettes addictive apart from nicotine. In a focus group analysis, we recently identified 3 unique features of e-cigarettes that users linked to experiences of addiction: the pleasant taste, unobtrusiveness, and unlimited usability of e-cigarettes.

**Objective:**

This study aimed to validate the previously identified features of e-cigarette addictive potential by triangulating data from the netnographic analysis and focus group discussions.

**Methods:**

Drawing on a netnographic analysis of 3 popular, German-language e-cigarette forums, we studied whether experiences of addiction were linked to specific e-cigarette features. We included 451 threads in the analysis that had been coded for addictive experiences in a previous study by our team. First, we conducted a deductive analysis with preregistered codes to determine whether the features of pleasant taste, unobtrusiveness, and unlimited usability were mentioned in relation to the addictive potential of e-cigarettes in the online forums. Second, an inductive approach was chosen to identify further possible addictive features of e-cigarettes.

**Results:**

Our deductive analysis confirmed that the features highlighted in our previous focus group study (pleasant taste, unobtrusiveness, and unlimited usability) were also frequently discussed in online forums in connection to addictive symptoms. In addition, our inductive analysis identified nicotine dosage as a significant feature linked to addiction. Users reported varying their nicotine doses for different reasons, leading to the identification of four distinct user types based on dosing patterns: (1) high doses for intermittent, (2) high doses for constant use, (3) low doses for constant use, and (4) switching between high and low doses depending on the situation.

**Conclusions:**

Our comprehensive analysis of online forum threads revealed that users’ experiences of addiction are linked to 4 specific features unique to e-cigarettes: pleasant taste, unobtrusiveness, unlimited usability, and nicotine dosage. Recognizing these addictive features of e-cigarettes is crucial for designing cessation programs and informing public health policies to reduce the addictiveness of e-cigarettes.

## Introduction

Electronic cigarettes, also known as e-cigarettes, are devices powered by batteries. They function by heating a liquid mixture, which usually includes nicotine and flavor, to produce an aerosol that users inhale [1]. e-Cigarettes are often used as a tool to quit smoking, with evidence showing that they are more effective than traditional nicotine replacement products [2]. However, many individuals who successfully quit smoking using these devices tend to continue vaping in the long term [2]. Indeed, research has indicated that e-cigarette users experience addiction, with studies revealing notable levels of nicotine dependence among e-cigarette users [3,4]. Moreover, the uptake of e-cigarettes among young people who have never smoked has risen [5], indicating that e-cigarettes are attractive beyond their use as smoking cessation aids. Thus, understanding the factors driving the attractiveness and addictiveness of e-cigarettes is crucial for improving prevention and cessation strategies.

In a comprehensive netnographic analysis of e-cigarette online forums, we previously examined self-reports of users in online forums using a netnographic approach. Some, but not all, users reported subjective experiences that met the criteria for a tobacco use disorder as classified by the fifth edition of the *Diagnostic and Statistical Manual of Mental Disorders-Fifth Edition* (*DSM-5*) [6]. In a further analysis of focus groups, we identified 3 unique aspects of e-cigarettes that were related to their addictiveness according to e-cigarette users: their pleasant taste, unobtrusiveness, and unlimited usability [7]. The aspect of pleasant taste has frequently been highlighted as the central component of the addictiveness of e-cigarettes. For example, mint flavor has been reported to be more addictive than other flavors by young adult e-cigarette users [8], while fruity flavors are perceived as less of a health risk [9]. The second unique characteristic, unobtrusiveness, describes the possibility of using the e-cigarette almost unnoticed without producing ash or unpleasant smells. This makes e-cigarettes more attractive compared with tobacco products [7]. Furthermore, since the user can control the size and density of vapor clouds, unobtrusiveness encourages using e-cigarettes even in places where it is not allowed to vape [10,11]. According to the results of our focus group study, this feature is associated with a higher frequency of use [7]. Likewise, the third e-cigarette characteristic identified in the focus groups, unlimited usability, was also associated with a high frequency of use and automaticity. Depending on their battery, tank size, and behavior of their user, e-cigarettes can be used for up to 20 hours or 800 puffs and more. Because they do not have to be lit like tobacco cigarettes, users can vape nonstop. The “ready availability” has also been identified by Kim et al [12] as a unique feature of e-cigarettes.

**Figure 1:**
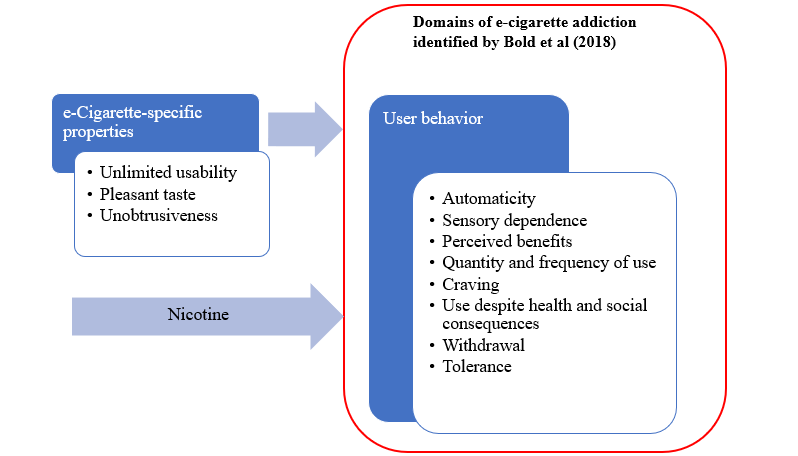
The model of the addictive potential of e-cigarettes (MAPE) is based on focus group interviews with e-cigarette users.

The 3 unique e-cigarette features are depicted in the MAPE (Model of the Addictive Potential of E-cigarettes) model (Figure 1 [7,13]). The development of the MAPE model was based on a qualitative approach in the form of focus group interviews [7]. Using diverse methods of data collection can significantly enhance the depth of understanding regarding the subject under investigation [14]. To validate the MAPE model, we opted for a netnographic approach, in which ethnographic techniques are applied to online social interactions [15]. An advantage of this approach is its capacity to mitigate various sources of bias prevalent in the field, such as selection, groupthink, interviewer, and social desirability effects [16]. In the discussion part of this paper, we will triangulate the findings of the qualitative and netnographic approaches to elaborate on what makes e-cigarettes attractive and addictive. A better understanding of what draws users to these products would support the development of strategies to prevent initiation among nonusers and to promote cessation. Finally, comprehending the addictive nature of e-cigarettes can inform public health policies aimed at reducing their appeal and mitigating long-term health risks.

## Methods

### Study Design and Setting

Since many e-cigarette users discuss their e-cigarette use in online forums [17,18], we chose netnographic analysis as the method for our analysis. Analyzing online discourse allows an understanding of the consumer’s perspective without the influence of the social desirability bias to please the researcher [16]. Especially sensitive topics, such as health status or addiction, are often discussed in the anonymous online setting [19], which makes the online setting appropriate for studying the addictive potential of e-cigarettes.

### Data Collection

**Figure 2:**
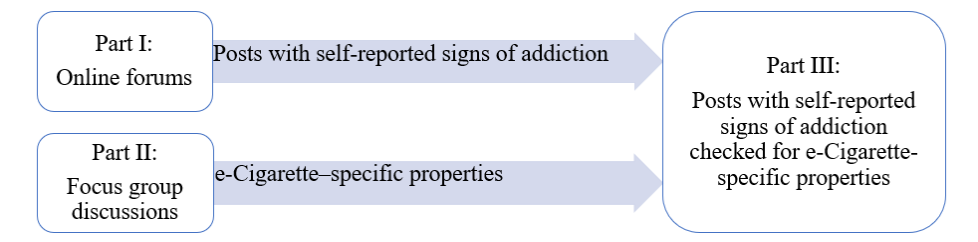
Overview of the research process to triangulate findings from netnographic analysis of e-cigarette online forums (study I) and focus group discussions with e-cigarette users (study II).

This study is based on results from 2 previous studies from the EVAPE (Evaluation of the Addictive Potential of E-cigarettes) project. In the first study posts in 3 large, German-speaking, e-cigarette online forums were scanned for addiction-specific keywords and subsequently extracted. The keyword search in all forums returned 5337 threads (database lock on April 9, 2021). Of the 5337 threads initially screened in the 3 forums, 451 contained relevant information regarding addiction and were included in the data analysis. Within these posts, self-reported signs of addiction were coded based on the *DSM-5* criteria for substance use disorder [6]. For a full description of data collection within the forums, please see our previous publication [6]. We found that users reported typical symptoms of addiction such as excessive time spent vaping, craving, and continued use despite health issues–related to e-cigarettes. In the second study of the EVAPE project, we conducted focus group discussions to identify e-cigarette–specific features related to addiction symptoms [7]. In 5 focus groups with 14 e-cigarette users overall, we asked users about e-cigarette–specific addiction symptoms. Analysis of the addiction experiences reported in the focus groups was based on the 10 domains of e-cigarette addiction previously identified by Bold et al [13]. We then inductively added codes for the e-cigarette properties that were identified as addictive by users. In this paper, we aimed to triangulate the results of the 2 studies and investigate whether the previously identified self-reported signs of addiction according to the MAPE model are linked to e-cigarette–specific features by the users of e-cigarette online forums (Figure 2 [6,7]).

### Data Analysis

We used the analysis software MAXQDA (VERBI Software) [20] for content analysis based on the approach by Clarke et al [21]. To evaluate the connection between e-cigarette–specific properties and perceived addiction symptoms, every code-identifying addiction symptom in our previous study [6] was evaluated again to check whether an e-cigarette feature was linked to the experience of addiction. The e-cigarette–specific properties from the MAPE model constituted the deductive coding system (Table 1). A post was only coded when the reported addiction symptom was linked to a specific e-cigarette feature. The deductive codes were registered beforehand on Open Science Framework storage [22]. Two authors (MA and NW) independently double-coded the posts.

In the second step, we used an inductive approach to identify e-cigarette–specific characteristics not covered by the MAPE model, namely, different user types based on the combination of nicotine dosage and frequency of use. Authors MA and NW agreed on the new code and also independently double-coded it. Agreement between the authors was then tested for all codes (deductive and inductive) using MAXQDA’s tool for intercoder agreement resulting in a code intersection rate of 87% at the segment level, indicating a substantial level of intercoder agreement. The authors discussed disagreements and, in the few cases of persistent disagreement, a third author was consulted (SS) to reach a consensus. Finally, all quotes were anonymized and forward-translated from German to English.

**Table 1:** Codebook for the deductive coding process of identifying e-cigarette properties linked to addictiveness in user posts.

e-Cigarette property	User description for coding
Unlimited usability	Users describe that unlimited usability leads to experiences of addiction symptoms. For example, long battery life, large tanks and not having to light up are indicators of the unlimited usability of e-cigarettes.
Pleasant taste	Users describe that the pleasant taste and smell of e-cigarette liquids lead to experiences of addiction symptoms. For example, favorite liquids could be discussed.
Unobtrusiveness	Users describe that unobtrusiveness leads to experiences of addiction symptoms. Features of unobtrusiveness include those e-cigarettes can be used almost everywhere even in places where it is prohibited (stealthing), they do not leave waste (eg, ash), and they do not leave an unpleasant smell.

### Ethical Considerations

Our study design was preregistered on the Open Science Framework and detailed in a previous publication to ensure transparency [22,23]. The Medical Ethics Committee of the Medical Faculty Mannheim at the University of Heidelberg approved our methodology for the netnographic and qualitative study (2017-567-N-MA). For the netnographic analysis, the forums’ terms and conditions informed users about the public nature of their posts. In addition, to protect user anonymity, we selected forums with a large number of active users and ensured no personal information was documented. This approach aligns with ethical guidelines for online research [24]. Participants in focus groups signed a consent form enabling us to use their anonymized data for research purposes.

## Results

### Deductive Codes

In the following, the deductive codes based on the MAPE model are listed.

#### Pleasant Taste

The pleasant taste of e-cigarette liquids was the characteristic discussed most frequently by users in relation to addiction. For example, users wrote about their favorite liquid or which liquids they enjoyed in which situations. Many users highlighted the taste as their favorite aspect of vaping, or “Taste- the holy grail of vapers!” as one user stated. Liquid taste was often mentioned as a reason to vape more, which led to self-reported craving in some users:

Sometimes the taste is so good - I just can’t get enough. Or I think about what I want to try next: another liquid or a mixture of the current and another one - so I have to use up my tank quickly.

Especially sweet flavors were mentioned as addictive and were sometimes compared to candy:

When I crave a specific sweet, I just choose the matching liquid. For example, when I want to eat chocolate, I vape chocolate liquid.

#### Unobtrusiveness

The fact that e-cigarette vapor does not leave a lingering smell and generates no waste, such as ash, was named as a reason for e-cigarette addictiveness. This characteristic was often compared with cigarettes:

I feel like I crave vaping constantly. I vape whenever possible. I enjoy vaping because there is no ash or smell in contrast to tobacco smoking.

The unobtrusiveness of e-cigarettes was also identified as one reason for e-cigarette use at home, at the office, or in the car:

When I was still smoking, I did not smoke in my flat because I find that disgusting. Now I vape in my flat and chain vape when I’m sitting in front of the TV.

This example suggests that the possibility of e-cigarette use at home also led to an increased frequency of vaping. Finally, not only e-cigarette users but also the people surrounding them seem to view vaping as less obtrusive than tobacco second-hand smoke. One user stated:

In the office, I vape non-stop because my colleague is not annoyed by that. So most of the time I have my e-cigarette in my mouth.

Again, this user behavior was often contrasted with previous experiences with tobacco smoking:

More and more often I think I vape too much. One disadvantage is that I can vape everywhere: At work, at home, visiting my friends. This was not the case when I was still smoking.

Some users also admitted to “stealthing”, a term used in the e-cigarette community to describe vaping in places where it is forbidden to vape or smoke.

#### Unlimited Use

e-Cigarette users mentioned the possibility of using their e-cigarettes for a long time as one reason for nonstop vaping and cravings. Unlimited use was often discussed in the context of improving this feature by buying new equipment. One user reported:

I was looking for hardware with long battery life because I had to recharge my former e-cigarette twice a day when vaping constantly. I found an e-cigarette model, which I only have to recharge after three days. So, I can vape constantly now.

The option to use e-cigarettes for a long period was also contrasted with previous cigarette smoking. Another user reported:

Imitating my former cigarette smoking is not possible, because there is not “one cigarette” to smoke. So now, I chain vape. And I don´t like that.

### Inductive Codes

One new code was identified during the inductive coding process: the ability to choose nicotine dosage in relation to craving.

#### Nicotine Dosage

**Figure 3:**
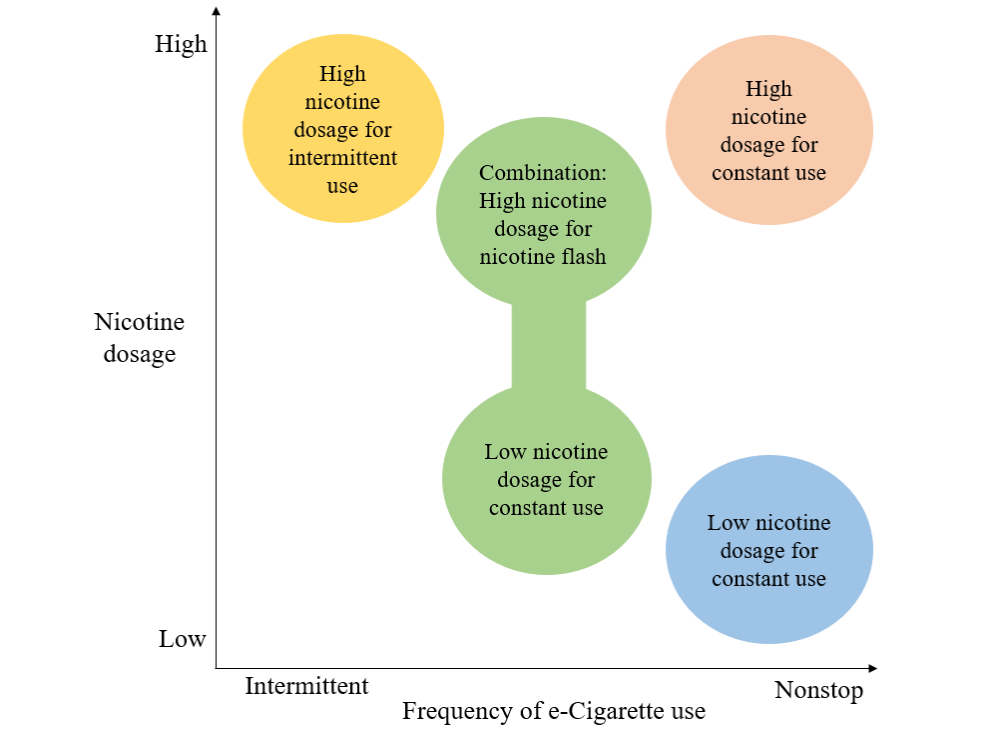
Visualization of 4 different user types identified in the inductive analysis of posts in e-cigarette forums.

The possibility of choosing liquids with user-specific nicotine doses or mixing their own liquids was a recurring topic in the forums. This feature was related to experiencing addiction and also trying to combat addiction symptoms. We identified 4 user types who choose their nicotine doses according to different rationales. A summary of the 4 user types is provided in Figure 3.

#### Low Nicotine Dosages for Constant Use

These users reported vaping e-cigarettes with low nicotine doses in order to be able to use their e-cigarettes nonstop:

At the beginning, I vaped 6 mg. For me as a chain-vaper, that was too much: I got dizzy. Nowadays 3 mg and everything is fine.

Typically, the nicotine doses mentioned by these users range from 1 mg to 5 mg. Often, users reported vaping lower doses to counteract negative health effects or make them more tolerable. For instance, one user posted:

At the end of the day I got a headache. I decided to dose down the nicotine in my e-cigarette, so I am able to vape constantly again.

Consequently, this code was often found in relation to the addiction symptom of e-cigarette use despite negative health effects.

#### High Nicotine Dosages for Intermittent Use

In contrast to users who use low nicotine doses for constant use, these users choose liquids with high nicotine doses in order to not vape constantly. For instance, one user reported, “I switched from 12 mg to 18 mg for controlling and reducing my chain-vaping.” Thus, this user type aims to control their usage behavior through higher doses.

#### High Nicotine Dose for Constant Use

These e-cigarette users regularly use very high nicotine doses to feel a nicotine flash. They often mentioned having developed a tolerance for lower nicotine doses, as this example demonstrates:

I started with 18 mg nicotine. That was not enough for me to get a flash, so I switched to 24 mg. I don’t want less nicotine. I have been vaping 24 mg in the last 3 years and I am happy with it.

Another user reported:

I vape 18 mg in the morning with my coffee and as a nicotine boost during the day (…). In the evening on the sofa I then switch to 9 or 12er fruity liquid - in almost continuous vaping mode.

Therefore, this user group combats tolerance symptoms with higher doses to satisfy their addiction.

#### High and Low Nicotine Dose in Combination

Some vapers also reported using an e-cigarette with a moderate nicotine dose in combination with a second one with a very high dose: “I have an emergency device with 18 mg. Ready for action.” Often, the different e-cigarettes are meant for different situations, as this quote exemplifies:

When I know there is no time for vaping, I put 18 mg in my (hardware), 15-18 Watt-4 puffs and I am done. By default, I use 9 mg and 15 Watt for chain-vaping.

The e-cigarette with higher nicotine concentration is thus used to experience a nicotine flash.

## Discussion

### Principal Findings

**Figure 4:**
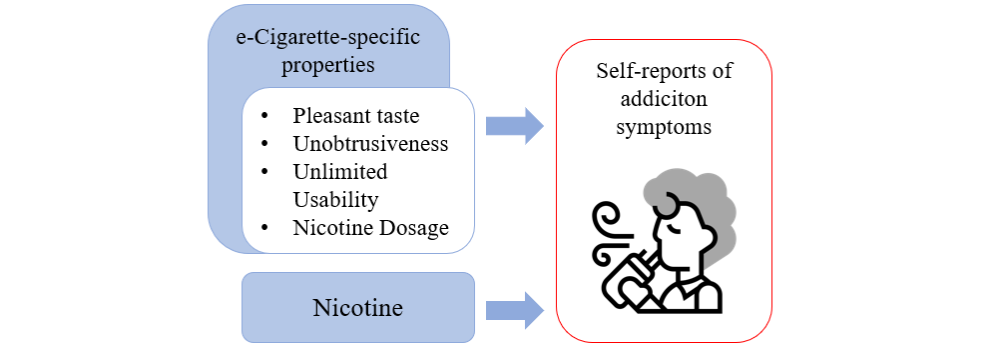
The updated model of the addictive potential of e-cigarettes is based on data triangulation.

In this study, using data triangulation, we showed that 3 specific characteristics of e-cigarettes identified in the MAPE model were also observed in e-cigarette online forums. The pleasant taste, unlimited usability, and unobtrusiveness of e-cigarettes were discussed by forum members in relation to addictive symptoms. Similarly to the focus groups previously conducted as part of the EVAPE project with e-cigarette users [7], users in the e-cigarette online forums reported that the 3 aspects highlighted in the MAPE model were attractive and thus addictive, therefore, validating the MAPE model in a different setting. Moreover, in contrast to the focus groups, the netnographic context allowed us to access user experiences firsthand without an interviewer bias. In addition, we were able to identify one further unique e-cigarette feature: nicotine dosage. The updated MAPE model is depicted in Figure 4.

### Triangulation

In addition to the 3 distinct features of e-cigarettes (pleasant taste, unobtrusiveness, unlimited use) that we previously identified in a focus group study, the netnographic analysis revealed one additional feature associated with e-cigarette addictiveness. In this study, nicotine dosage emerged as a prominent e-cigarette feature frequently discussed in relation to addiction. The analysis of forum discussions on dosage revealed the existence of 4 distinct patterns among users. Some users reported opting for low nicotine doses in order to use their e-cigarettes continuously, while a second group preferred high doses to reduce the frequency of vaping. In a laboratory study, Dawkins et al [25] compared the user behavior of their participants on 2 different days, being given an e-cigarette with high (24 mg/mL) one day and low (6 mg/mL) nicotine concentration liquids the other day. Vaping the low nicotine concentration, users puffed more frequently, longer, and nearly doubled the amount of liquid consumed. This is in line with user reports analyzed in our study, showing that low nicotine doses were related to constant use. However, our analysis extends beyond controlled laboratory settings, revealing that vapers actively select low or high nicotine doses depending on the user behavior they prefer. It is possible that there are different motives behind the usage behaviors. The fact that one user group likes to use their e-cigarette nonstop, but higher nicotine doses lead to unpleasant effects, could indicate that these users are motivated by psychological dependence, for example, habituation or sensory aspects (throat hit, stimulation of the respiratory tract), rather than a purely physiological dependence on nicotine. It is already known that nonpharmacological aspects of tobacco cigarette smoking reduce the urge to smoke even more effectively than the direct pharmacological effect of nicotine alone [26,27]. Conversely, people who use high nicotine concentrations but vape less may be driven more by nicotine intake or physiological withdrawal symptoms. Our study indicates that vapers who use high nicotine concentrations, self-titrate to control addiction symptoms, meaning they adjust their nicotine intake either by different use behavior or by adjusting nicotine levels. This behavior helps to effectively manage craving and withdrawal symptoms to reduce constant vaping [28].

Furthermore, we identified 2 groups of forum users, who used liquids with very high nicotine concentrations to experience a nicotine flash. One group exhibited symptoms indicative of nicotine tolerance, leading them to consistently vape liquids with high nicotine doses in order to experience a nicotine flash. This is in line with a retrospective survey study by Browne and Todd [29], who found that e-cigarette users who have been vaping for a longer time used a higher nicotine dosage than short-term users. Longitudinal studies of nicotine dosing changes over time could provide further insights into this pattern. In contrast, another group used liquids with high nicotine concentrations next to lower-dosed liquids to experience a nicotine flash. This was done in specific situations that were perceived as stressful or in which nonstop vaping was not possible. For dual–nicotine format users, Perry et al [30] observed that craving for tobacco cigarettes compared with e-cigarettes increased in stressful situations. Our study indicates that single–nicotine format users experienced cravings in such situations and equip themselves with an “emergency” high nicotine dose device to alleviate their withdrawal symptoms. Earlier studies have shown that many smokers mistakenly believe cigarettes reduce stress when in reality, smoking only alleviates nicotine withdrawal symptoms [31]. Thus, some vapers may perceive high-nicotine liquids as more effective for stress relief because they are more effective at reducing withdrawal symptoms, resulting in a temporary improvement in mood and state of mind, but a long-term increase in stress levels.

The aspect of nicotine dosage and its relation to addiction is not yet well understood in e-cigarette users. A review of 104 studies indicates that increased nicotine concentrations are linked to heightened abuse potential and appeal [32]. While some studies compare puff durations between e-cigarette users [33], no study has yet investigated differences and rationales for self-chosen nicotine concentration. Our analysis implies that e-cigarette users select nicotine doses according to their preferred use behavior. For example, some users use high-nicotine e-cigarettes occasionally for a quick nicotine flash, while others use low-nicotine e-cigarettes more frequently throughout the day. In turn, some users have multiple devices for different situations and choose between them depending on their current nicotine craving or social context. This is consistent with the findings of a previous study of puff topography in natural environments [34]. User reports collected here suggest that the opportunity to select nicotine dosage might thus be a pathway to facilitating nicotine dependency and tolerance. Given the diverse rationales observed in individuals’ choice of nicotine doses and the indications of self-titration found in this study, future research should delve deeper into the relationship between self-chosen nicotine concentration and addiction in e-cigarette users.

### Implications for Practice

The updated MAPE model can help inform both preventive and cessation programs for e-cigarette users. Our research demonstrated that the pleasant taste of e-cigarettes is a crucial factor for the enjoyment of vaping and related addiction. Accordingly, banning certain liquid flavors might be an option to reduce the attractiveness of these products. Currently, 6 countries ban e-cigarette liquids with flavors other than tobacco to make vaping less attractive for young people [35]. Notably, in Germany, where our study was conducted, such a ban is not currently in place. Our data suggest that implementing a ban on liquid flavors might mitigate the addictive potential of e-cigarettes, but evidence is currently lacking in reports on the effects of such a ban [36]. Evidence from other countries indicates that a ban on flavored e-cigarettes alone may increase the consumption of tobacco cigarettes or other flavored electronic nicotine delivery systems that are exempt from the restrictions, such as disposable e-cigarettes [37]. In addition, most respondents appeared to continue to use banned flavors despite the restrictions [38]. This illustrates that the effectiveness of such regulations should not be considered independently of other tobacco or nicotine products and their user groups. Since Germany has weak tobacco regulations overall [39], tobacco control should be strengthened on all devices to effectively safeguard public health.

In addition, the ability to use e-cigarettes for an extended duration was associated with increased usage frequency and automatic use, indicative of addiction. For individuals seeking to quit or reduce vaping, physicians could be advised to recommend the use of a puff counter, a device that displays the number of puffs taken within a specific timeframe. In light of our findings, such a device could assist users to regain control over their e-cigarette consumption. The other unique aspects of e-cigarettes (unobtrusiveness and the ability to control nicotine dosage) are more challenging to regulate. These features make it difficult for regulatory measures to effectively address these aspects of e-cigarette use. The different user preferences for nicotine doses found in this study also highlight that this aspect needs to be further investigated to better understand the association between nicotine dosage, duration of use, and the development of addiction.

### Limitations

An in-depth discussion of applying a netnographic approach to e-cigarette online forums can be found in a previous paper by Szafran et al [6]. In brief, applying a netnographic approach potentially mitigates biases associated with personal or group interviews, such as interviewer or social desirability biases [40]. Nonetheless, a bias might still remain, as research has shown that forum users selectively disclose information to project a favorable image of themselves to their peers [41]. In addition, our dataset encompasses a specific subgroup of e-cigarette users whose overall positive outlook toward e-cigarettes may have influenced the underreporting of addiction symptoms. In addition, we cannot completely exclude the possibility that comments may have been influenced by tobacco company marketing communications, although this is prohibited by the forum’s terms of use. Furthermore, we used terms derived from *DSM-5* criteria for tobacco use disorder to search for user posts in the online forums, thus e-cigarette–specific addiction symptoms such as automaticity may not have been included.

Another limitation of this research approach is that we have no data on user characteristics, such as age or gender, as well as usage characteristics, such as frequency of consumption or total consumption. This also applies to the potential inclusion of posts from dual users, who use both e-cigarettes and tobacco. While we attempted to exclude posts explicitly mentioning dual use, it was impossible to filter out all such content as it often was not reported. Previous research has shown that dual users tend to exhibit higher levels of addiction compared with exclusive e-cigarette users [42], which should be factored into result interpretation. Furthermore, we could not distinguish between users who used nicotine-containing liquids from those who did not, and in cases where nicotine was used, we lack data on nicotine concentration in the liquids used. This was not true for the codes on dosage, however, where users shared specific nicotine doses with each other.

### Conclusion

In the MAPE model, we previously summarized the distinctive aspects that make e-cigarettes addictive: their pleasant taste, unobtrusiveness, and unlimited usability. This netnographic analysis enhances the MAPE model by incorporating insights from e-cigarette online forums, revealing the additional dimension of nicotine dosage. The patterns of nicotine dosage, for example, low concentrations for continuous use or high concentrations for intermittent use, reflect the different rationales among users.

These findings have important implications for policy and practice. The role of e-cigarettes’ pleasant taste for experiences of addiction highlights the potential role of flavor bans to mitigate addiction. The association between unlimited use and self-reported addiction suggests the potential utility of puff counters in cessation efforts.

In essence, our study contributes a nuanced understanding of what makes e-cigarettes addictive, emphasizing user perspectives on e-cigarettes’ unique features. This qualitative exploration also highlighted the need to further explore user rationales for nicotine dosage.

## Abbreviations

*DSM-5*: *Diagnostic and Statistical Manual of Mental Disorders Fifth Edition*
EVAPE: Evaluation of the Addictive Potential of E-cigarettes
MAPE: Model of the Addictive Potential of E-cigarettes

## Data Availability

The datasets generated during and/or analyzed during this study are not publicly available to ensure user anonymity, but further details may be available from the corresponding author on reasonable request.
